# Simplified Method of Microcontact Force Measurement by Using Micropressure Sensor

**DOI:** 10.3390/mi12050515

**Published:** 2021-05-04

**Authors:** Huamin Zhu, Fuzhong Zheng, Huiwen Leng, Cheng Zhang, Kun Luo, Yibo Cao, Xing Yang

**Affiliations:** 1 The State Key Laboratory of Precision Measurement Technology and Instrumentation, Department of Precision Instrument, Tsinghua University, Beijing 100084, China; zhuhm@bhqditi.com (H.Z.); yibocao95@gmail.com (Y.C.); 2 Beihang-Goertek Joint Microelectronics Institute, Qingdao Research Institute, Beihang University, Qingdao 266000, China; 3 College of Mechanical Engineering, Sichuan University of Science and Engineering, Zigong 643000, China; 4 School of Electromechanical and Automotive Engineering, Yantai University, Yantai 264005, China; lenghw86@126.com (H.L.); 18363827697@163.com (K.L.); 5 College of Mechanical and Electrical Engineering, Wenzhou University, Wenzhou 325035, China; zhangcheng@wzu.edu.cn

**Keywords:** micropressure sensor, microcontact force measurement, finite element simulation

## Abstract

Microcontact force measurement is widely applied in micro/nano manufacturing, medicine and microelectromechanical systems. Most microcontact force measurements are performed by using mass comparators, nano-indenter and precision electronic balance, and weighing sensors. However, these instruments have a complex structure and high cost. Nevertheless, the rapid development of microsensor technology provides a new, simple and low-cost approach for microcontact force measurement. In this study, we present a method of microcontact force measurement by using micropressure sensors and study the relationship amongst the microcontact force, output voltage and contact position of the sensor. We use a microcapacitance pressure sensor as an example, then we perform a simulation calculation and construct a microcontact force experiment system to verify the simulation results. The experimental and simulation results are consistent. In addition, an equation that describes the relationship amongst the microcontact force, output voltage and contact position of the sensor is obtained. Based on this simple and low-cost method, we build a micro-manipulation system, which indicates that the micropressure sensors can be used to measure microcontact force in various applications easily and cost-effectively. Furthermore, it is considerably relevant to research and application in this field.

## 1. Introduction

Microforce measurement has been extensively used in micro/nano manufacturing, biomedical engineering [1,2], instrumentation, electronic power, aerospace, precision control [3,4], and other fields [5]. For example, spotting is a key step in biochip technology. The value of spot contact force is at level of the micro-Newton, and its monitoring and control are key factors to ensure spot quality [6]. For another example, microcapsules are required to undergo microsize compression to select suitable materials and design structures for transporting drugs, enzymes, or living cells. In these examples, microcontact force monitoring is one of the keys [7]. Furthermore, in many other fields, such as new materials, microelectromechanical system (MEMS) technologies, microinspection, characteristic research, friction observation and force analysis, the demand for microcontact force measurement has been increasing [8]. At present, various methods are available for microforce monitoring. For example, the electrostatic balance bar developed by the National Institute of Standards and Technology is used as a precision coaxial cylindrical capacitor to produce the known value of electrostatic force. This bar achieves microcontact force values and accurate measurements based on the lever principle and the electrostatic recurrence principle [9,10,11]. A horizontal swing electrostatic force device designed by Physikalisch–Technische Bundesanstalt uses nine parallel plates as electrodes and eight electrodes corresponding to two external conductive disks. The device can measure static forces below 10^−5^ N and has a resolution of 10^−12^ N [12,13,14]. On the basis of the measurement principle of atomic force microscopy [15,16], Korea Research Institute of Standards and Science produces a nanometer force standard instrument, which combines precision balance with an atomic force microcantilever and has a resolution of 1 nN precision balance and a resolution of 1 nm precision translation table, for microforce measurement [17]. The ultramicro balance developed by METTLER TOLEDO (USA) can achieve a resolution of 0.1 μg and measure contact force below 1 nN. Although the measurement resolution of existing microcontact force monitoring methods and devices can reach nN or even pN level, most of them involve complex instruments and relatively strict experimental conditions. These factors limit the wide application of microforce measurement in scientific research, production, and other fields to some extent. Therefore, studying a simple and low-cost method for measuring microcontact force is crucial.

The development of MEMS and nanotechnology has allowed the mass fabrication of micropressure sensors with low cost, high precision, good consistency, small volume and mature technology. These sensors can be categorised in accordance with their measurement principle, as follows: piezoresistive, capacitive, piezoelectric and resonant sensors [18]. These sensors may provide a new and simple method for microcontact force measurement. Recently, research on the relationship amongst the microcontact force, output voltage, and contact position of the sensor is still being proposed and updated [19,20,21]. However, the relationship that the capacitance between two diaphragm plates changes with the concentrated force and its relative position caused by a small-tip probe on a circular thin plate has not yet been extensively studied. In this study, we conduct a theoretical analysis of a capacitive micropressure sensor that suffers from contact force. Then, we construct a finite element model and conduct simulation calculation. A microcontact force experiment system is developed to verify the simulation results. Lastly, we obtain an experimental fitting formula of microcontact force measurement based on the micropressure sensor; this formula can describe the relationship amongst the microcontact force, output voltage and contact position of the sensor. This microcontact force measurement method based on micropressure sensors has some advantages, including simple structure, low application threshold, wide application fields, and low cost.

## 2. Principle and Method Analysis

**Figure 1:**
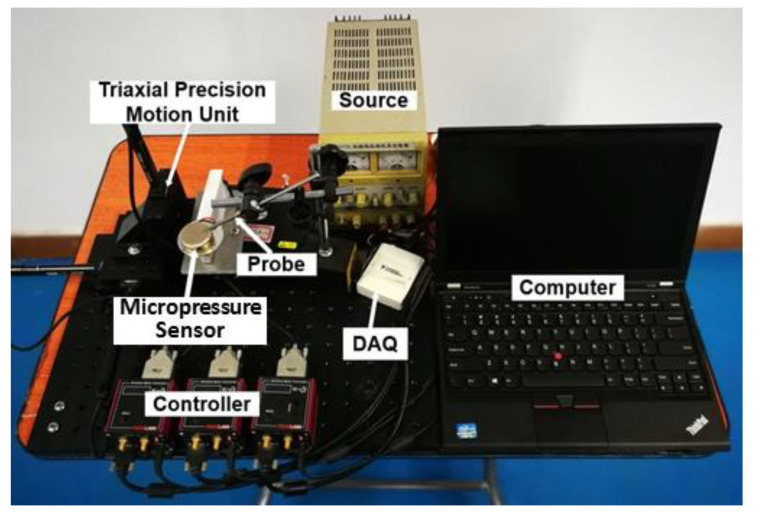
Microcontact force monitoring system based on a micropressure sensor.

Pressure sensors are mainly used for liquid or gas pressure measurement [22]. However, when the pressure sensor is used to measure solid contact force, the situation becomes different from the preceding conditions. To monitor microcontact force, a manipulation system is established on the basis of a micropressure sensor (Figure 1). The system is mainly composed of a capacitive micropressure sensor, a voltage source, a triaxial precision motion unit, a computer, a data acquisition (DAQ) card, a probe, and controllers. When the probe contacts the sensing surface of the pressure sensor for manipulation, the contact force action point can be determined in accordance with the moving distance of the precision stage. As the response time of the sensor is less than 1 ms, the microcontact force acting on the micropressure sensor is almost in real time. The capacitive micropressure sensor (CCPS32, KAVLICO, Thousand Oaks, CA, USA) we used in the experiment has the following outstanding features. The capacitance of the substrate electrode and the diaphragm electrode changes in proportion to the pressure. The built-in temperature sensor continuously measures the temperature of the medium and compensates the temperature. The standardized high output has strong anti-interference ability, and the sensor has high temperature stability and time stability, with temperature compensation of −20 to 80 °C. The changes of capacitance are finely tuned by the laser, and the special signal conditioning circuit of the sensor amplifies and outputs a DC voltage of up to 4000 mV.

**Figure 2:**
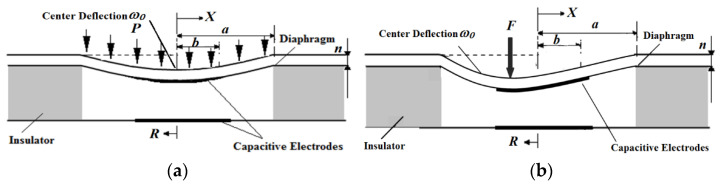
(**a**) Thin circular plate model with uniform load. (**b**) Thin circular plate model with concentrated force.

The structure and working principle of a capacitive pressure sensor is shown in Figure 2, where *R* is the polar radius, *X* represents the x-axis of the Cartesian coordinate system for the parallel of the membrane, *a* is the cavity radius of the circular capacitor, and *b* is the radius of the circular electrode plate. When the diaphragm is deformed under the action of liquid or gas pressure *P*, the distance between the film and the fixed electrode is changed, resulting in the change of capacitance between the upper and lower electrodes. The capacitance is converted into a voltage signal by the measuring circuit. Therefore, the measured pressure *P* can be obtained by monitoring the output voltage. According to the preceding working principle analysis, when the capacitive pressure sensor measures liquid or gas, its model could be equivalent to a thin circular plate that suffered from uniform load (pressure), as shown in Figure 2a.

As shown in Figure 2, the capacitance between the two parallel plates of the capacitive pressure sensor can be expressed by the following formula:

(1) $$C=\frac{\varepsilon_{0}\varepsilon_{r}A}{d}$$

where *ε*_0_, *ε_r_*, *A* and *d* are the permittivity of the vacuum, the relative permittivity of the medium between the two plates, the effective plate area and the distance between the two plates, respectively. According to Formula (1), when the effective plate area is fixed, the capacitance *C* between the two parallel plates is inversely proportional to *d*. When the diaphragm is subjected to the uniform load *P*, the deflection equation of the diaphragm is as follows [22]:

(2) $$\omega =\frac{P}{64D}{\left(a^{2}-r^{2}\right)}^{2}$$

where $D=\frac{En^{3}}{12\left(1-\nu^{2}\right)}$; *a* and *n* are the radius and thickness of the diaphragm, respectively; *r* is the polar radius; *E* is the Young’s modulus of the diaphragm material; *P* is the pressure acting on the upper surface of the diaphragm; and *ν* is the Poisson’s ratio. The deflection of the diaphragm is the largest at the center and decreases with increasing distance from the center. If the radius of the effective circle area of the plate is b, capacitance can be obtained by integrating the deflection equation of each position of the diaphragm through Equation (3) [22].

(3) $$C=\varepsilon_{0}\varepsilon_{r}\int_{0}^{2\pi }\int_{0}^{b}\frac{r}{d-\omega (r)}drd\theta$$

(4) $$C=8\pi \varepsilon_{0}\varepsilon_{r}\sqrt{\frac{D}{Pd}}\tanh^{-1}\left(\frac{8b^{2}\sqrt{PdD}}{64Dd-Pa^{2}(a^{2}-b^{2})}\right)$$

Equation (4) reveals a one-to-one relationship of pressure *P* and capacitance *C*, which exists between the two parallel plates; this relationship is the measurement principle of the capacitive pressure sensor.

As shown in Figure 2b, when the pressure sensor is used to measure the solid contact force, the situation varies from the situation where it is used to measure the gas or the liquid pressure, and the micropressure sensor diaphragm receives a concentrated contact force at a certain position. This equivalent mechanical model can be simplified as a circular thin plate suffers from a concentrated force. So far, research on the relationship between the deformation of a circular thin plate subjected to concentrated forces and the capacitance of the capacitive micropressure sensor is lacking. Therefore, this study focuses on building the mechanical model and derives the formulas.

Let’s introduce the following dimensionless parameters [23,24]:

(5) $$x=\frac{r^{2}}{a^{2}}$$

(6) $$y=\frac{{\left[3(1-\nu^{2})\right]}^{1/2}\omega }{n}$$

(7) $$v=\frac{dy}{dx}$$

it can be obtained from Equation (6):

(8) $$dy=\frac{{\left[3(1-\nu^{2})\right]}^{1/2}}{n}d\omega$$

On account of elastic characteristic, the solution in paper [21,22] is

(9) $$v=-{\left(\frac{2}{9}F\right)}^{1/3}{\left[x\;+\;\frac{1-3\nu }{3(1+\nu )}\right]}^{-2/3}$$

When the centre of the sensor’s diaphragm bears the concentrated load *F*, the diaphragm deflection can be obtained from Equations (5), (7)–(9):

(10) $$\omega =\frac{-3n{\left(\frac{2}{9}F\right)}^{1/3}}{{\left[3(1-\nu^{2})\right]}^{1/2}}{\left(\frac{r^{2}}{a^{2}}+\frac{1-3\nu }{3(1+\nu )}\right)}^{1/3}$$

(11) $$C=2\pi \varepsilon_{0}\varepsilon_{r}\int_{0}^{b}\frac{r}{d+\frac{3n{\left(\frac{2}{9}F\right)}^{1/3}}{{\left[3(1-\nu^{2})\right]}^{1/2}}{\left(\frac{r^{2}}{a^{2}}+\frac{1-3\nu }{3(1+\nu )}\right)}^{1/3}}dr$$

**Figure 3:**
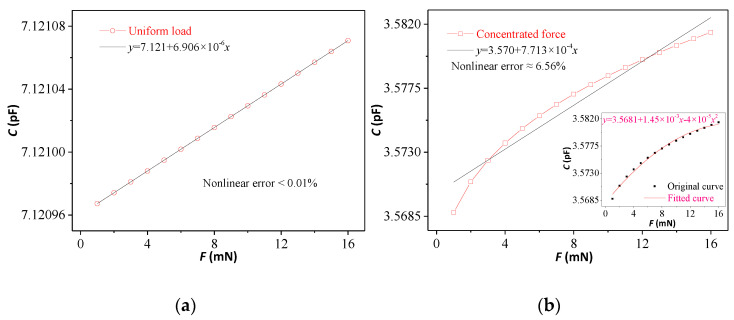
Calculation results by MATLAB (**a**) Thin circular plate model with uniform load. (**b**) Thin circular plate model with concentrated force.

Equation (11) shows that a nonlinear relationship exists between the concentrated force *F* and the capacitance *C*, *ε*_0_, *ε_r_*, *D*, *d*, *a* and *b* are fixed parameters; thus, the microcontact force *F* can be obtained by monitoring of the capacitor *C*, which is the principle of the microcontact force measurement using capacitive pressure sensors. Using the parameter values in Table 1 and MATLAB software, Equations (4) and (11) were solved, respectively. Then, Figure 3a,b could be obtained to show that the linear relation of *C* and *F* with nonlinear error <0.01% under uniform load and nonlinear error ≈6.56% under concentrated force. The inset of Figure 3b shows that a second order polynomial can fit well, but the coefficients of independent variable are very small. When the concentrated force was small and varied only in a small range, *C* could be considered to vary approximately linearly with *F*.

However, when the diaphragm suffers from the concentrated load outside the center, the calculation of the deflection and the capacitance becomes more complex. Under this condition, COMSOL finite element simulation software was used in this study to model and calculate the capacitance between the two parallel plates of the sensor when the concentrated load was applied to different positions of the sensor’s diaphragm.

**Figure 4:**
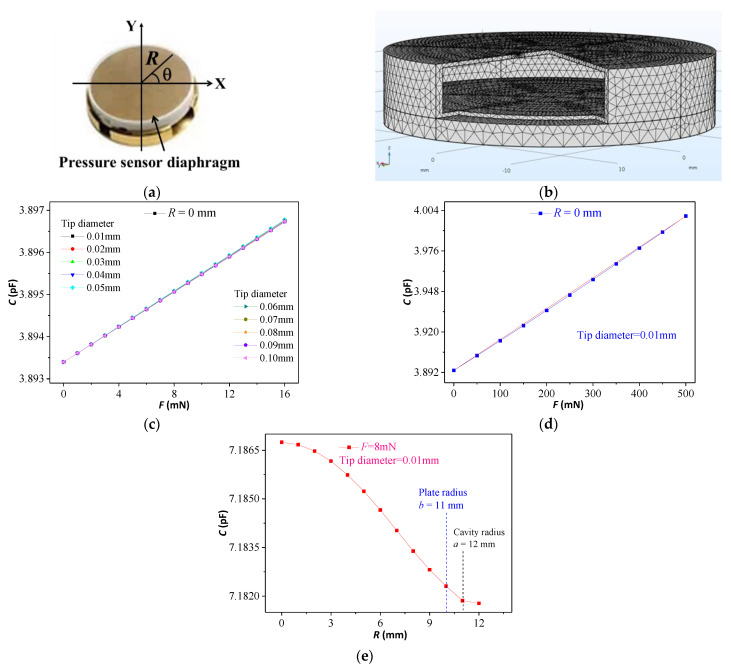
(**a**) Photo of the microcapacitive pressure sensor. (**b**) 3D geometry model of the pressure sensor. (**c**,**d**) Relation between the contact force of two different intervals and the capacitance of the pressure sensor with the plate radius *b* = 11 mm. (**e**) Relation between the radius of the contact force’s action point and the capacitance.

On the basis of the above analysis, the real capacitive sensor, as shown in Figure 4a, was split out of its core sensitive unit to establish a 3D geometry model for simulation analysis by software COMSOL. The model in Figure 4b is a figure after size scale adjustment, which is conducive to see the full view and local structure simultaneously. Coupling simulation of multiple physical field simulation can help us to study the following three cases: (1) Different concentration forces *F* (0~16 mN) and tip diameters (0.01~0.1 mm) were applied to a fixed position on the pressure sensor’s diaphragm. Related simulation results show that different positions along the radial direction have the same characteristics. (2) The simulation results in Figure 4c shows that an approximate linear relationship is evident between the contact force and the capacitance of the pressure sensor, and that the effect of tip diameter is not obvious in a certain range. So a fixed tip diameter 0.01 mm was used in the following simulations. Figure 4d shows the linearity is approximate while the bottom contact force is less than 500 mN. (3) The relation between the cavity radius *a* and the output capacitance was studied. The simulation results are shown in Figure 4e, where a nonlinear relationship is observed between the micropressure sensor’s capacitance and cavity radius of the contact force’s action point.

## 3. Experiments and Results

**Figure 5:**
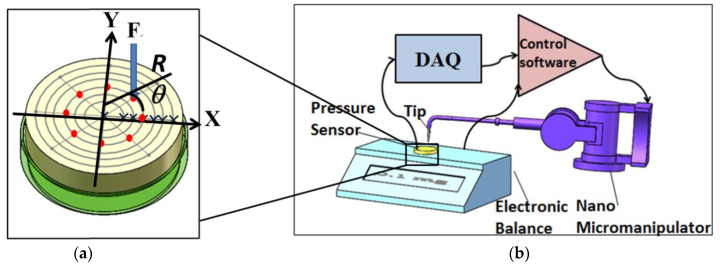
(**a**) Schematic of the contact force acting on the pressure sensor’s diaphragm. (**b**) Schematic of the experimental system.

**Figure 6:**
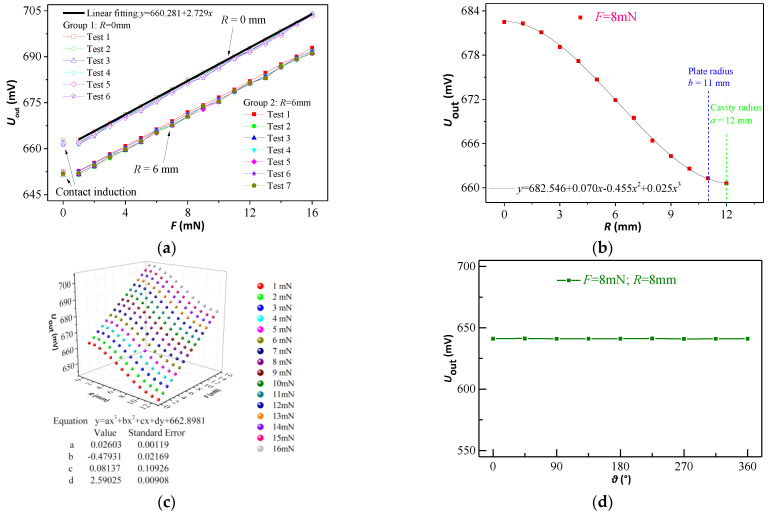
The tip diameter is 0.01 mm in all tests. (**a**) Experimental curve of the contact force vs. the output voltage. (**b**)Experimental curve of the polar radius of the contact force action point vs. the output voltage. (**c**) Fitting of the curve surface. (**d**) Experimental curve of the polar angle of the contact force action points vs. the output voltages.

A microcontact force experiment system was constructed to verify the theoretical formula and simulation results and to study the influence of force’s position and value on the output of the sensor when it is under the action of microcontact force. An internal circuit in the capacitive micropressure sensor was used in the experiment; this circuit could linearly convert the output capacitance signal to a voltage signal to facilitate measurement and practical use. Figure 5a presents a schematic of the contact force acting on the pressure sensor’s diaphragm. The contact force points were marked by red dots on the surface of the sensor. The measurement results are shown in Figure 6d, which means the structure of the sensor is symmetrical. Figure 5b shows a schematic of the experimental system, which comprises an electronic balance, the pressure sensor to be tested, a tungsten needle, a micro/nano manipulator, control software, and DAQ card. The ultraprecision electronic balance (METTLER TOLEDO XS205DU), which can weigh a maximum of 220/81 g objects and has a resolution of 0.1/0.01 mg. For the nano-manipulator (Kleindiek Nanotechnik MM3A), the step precision can reach 0.25 nm. Thus, in this experimental system, precise positioning could be realized, and tiny contact force ranging from 0.1 μN to 2.2 N could be applied to each position of the sensor diaphragm.

Using the experimental system, we experimentally studied the previously mentioned simulation results of finite element. Figure 6a shows that the concentrated forces by a small-tip probe correspond to analog voltage linear outputs. There are two groups of curves and each group has six curves. The two groups of curves were obtained at the position of *R* = 0 and 6 mm, respectively. When the tip of the probe contacts the sensing diaphragm, there will be an inductive output. Figure 6b shows the relationship between the analog voltage output and the cavity radius of the contact force’s action point. Their nonlinear characteristic is similar to the curve in Figure 4e. Then, the experimental data combined with simulation fitting results to fit the curve surface by using the software Origin. Figure 6c shows that the curve surface satisfied the following third-order equation:

(12) $$U=0.026R^{3}-0.479R^{2}+0.081R+2.590F+682.546$$

where *F* is the contact force (mN), *R* is the polar radius of the contact force action point (mm), and *U* is the output voltage of the pressure sensor (V). Figure 6d shows that the circular structure of the sensor is symmetrical inside, and confirms that the derivation of equation, the model of simulation and the test of experiment are believable.

From the above experimental and simulation results, we obtain the following conclusions: (1) When the microcontact force’s position on the pressure sensor diaphragm is fixed, the sensor’s output voltage with the contact force’s value shows a linear relationship. (2) The output voltage of the sensor shows a one-to-one relationship with the position and value of the contact force. (3) The measured contact force can be obtained from the position of the contact force acting point and the output voltage. Thus, microcontact force can be measured using a pressure sensor.

The performance indicators (including resolution, range and stability of the proposed method are analysed. The resolutions in the different positions of the micropressure sensor’s sensing surface vary, i.e., decreasing with the increase in polar radius *R* In the centre of the sensor’s sensing surface, the resolution is the highest, i.e., approximately 100 N. The larger the polar radius *R* from the contact force point to the diaphragm centre is, the lower the resolution is. Moreover, as the sensing structure is symmetrical, the polar angle *θ* has no effect on resolution. The resolution could be described by Equation (13).

(13) $$F=0.010R^{3}+0.185R^{2}+0.031R+0.1$$

By using this pressure sensor to measure the microcontact force, the range of measured contact force may reach 1 N. Certainly, users can select the micropressure sensor with a suitable range and resolution to measure microforce according to their requirements. In addition, the data sheet of the capacitive micropressure sensor adopted in this method shows that it has a small stability less than 0.1% FS/year. Here, we use coefficient of variation to assess the repeatability of the measurement system, which is also known as the ratio of standard deviation and whose value is the standard deviation divided by the average value. The repeatability calculated from the experimental data is less than 0.1% FS.

## 4. Conclusions

This study presented a simplified and low-cost microcontact force measurement method based on micropressure sensors. The corresponding mechanical model and theoretical formula were investigated when microcontact forces were applied to the different positions of the pressure sensor’s diaphragm. Simulation analysis was also conducted to determine the relationships amongst the value, contact force position, and output capacitance of the micropressure sensor. Moreover, a microcontact force experiment system was constructed to verify the theoretical analysis and simulation results. The experimental and simulation results were consistent, verifying the feasibility of the proposed method. In addition, a formula was fitted on the basis of the experimental data for the expedient use of this method. Lastly, a probe microcontact force measurement system based on the proposed method was built, and some performance indicators, including resolution, range, and stability (i.e., 100 μN, 1 N, and 0.1 FS%, respectively) were analysed. Microforce measurement of various ranges and resolutions can be conveniently achieved by selecting a suitable pressure sensor. Therefore, the proposed method has some advantages, such as simplicity and low cost; the method could be used to measure microcontact force in various applications and has considerable relevance to research and application in the fields of instrumentation, micro/nano manufacturing, biomedicine, and precision control.

## Figures and Tables

**Table 1:** Sensor’s structural parameters used for MATLAB calculation and COMSOL simulation.

Parameter Name	Parameter Symbol	Value
Absolute dielectric constant	*ε*_0_	8.854187817 × 10^−12^ F/m
Air’s relative dielectric constant	*ε*_r_	1
Cavity radius of capacitor	*a*	12 mm
Plate radius of capacitor	*b*	11 mm
Distance between the two plates	*d*	0.5 mm
Thickness of the diaphragm	*n*	0.015 mm
Young’s modulus of the diaphragm	*E*	2.8 × 10^11^ Pa
Poisson’s ratio of the diaphragm	*ν*	0.33

## References

[1] Kim M.S., Pratt J.R. (2010). SI traceability: Current status and future trends for forces below 10 microNewtons. Measurement.

[2] Wang S., Ding J., Yun J. A Robotic System with Force Feedback for Micro-Surgery. Proceedings of the 2005 IEEE International Conference on Robotics and Automation.

[3] Yin Y., Zhou C. (2006). Optimal design of micro-force sensor for wire bonding with high acceleration and frequent movement. Sens. Actuators A Phys..

[4] Haddab Y., Chen Q. (2009). Improvement of strain gauges micro-forces measurement using Kalman optimal filtering. Mechatronics.

[5] Liu Z. (2012). Research on the Method of Micro-Force Measurement and Tracing Based on Lever. Master’s Thesis.

[6] Li J.G., Huo R.L. (2007). Biochip microarrayer. Mod. Sci. Instrum..

[7] Kim K., Cheng J. (2008). MEMS capacitive force sensors for micro-scale compression testing of biomaterials. MEMS.

[8] Lundstrom T., Clark W., Jalili N. (2017). Development of a novel precision instrument for high-resolution simultaneous normal and shear force measurements between small planar samples. Rev. Sci. Instrum..

[9] Newell D.B., Kramar J.A. (2003). The NIST microforce realization and measurement project. IEEE Trans. Instrum. Meas..

[10] Steele J.M.A. (1964). Conference on precision electromagnetic measurements. J. Sci. Instrum..

[11] Kramar J.A., Newell D.B. (2002). NIST electrostatic force balance experiment. Vdi Berichte.

[12] Schlegel C., Slanina O. (2012). Construction of a standard force machine for the range of 100 μN–200 mN. Measurement.

[13] Behrens I., Doering L. (2003). Piezoresistive cantilever as portable micro force calibration standard. J. Micromech. Microeng..

[14] Nesterov V. (2007). Facility and methods for the measurement of micro and nano forces in the range below 10−5 N with a resolution of 10−12 N (development concept). Meas. Sci. Technol..

[15] Kim M.S., Choi I.M., Park Y.K. (2007). Atomic force microscope probe calibration by use of a commercial precision balance. Measurement.

[16] Cumpson P.J., Clifford C.A., Hedley J. (2004). Quantitative analytical atomic force microscopy: A cantilever reference device for easy and accurate AFM spring-constant calibration. Meas. Sci. Technol..

[17] Kim M.S., Choi J.H., Park Y.K. (2006). Atomic force microscope cantilever calibration device for quantified force metrology at micro- or nano-scale regime: The nano force calibrator (NFC). Metrologia.

[18] Li W. (2005). A Study on the Design and Fabrication of MiCro Pressure Sensors. Master’s Thesis.

[19] Jindal S.K., Mahajan A., Raghuwanshi S.K. (2019). An inductive-capacitive-circuit-based micro-electromechanical system wireless capacitive pressure sensor for avionic applications: Preliminary investigations, theoretical modelling and simulation examination of newly proposed methodology. Meas. Control.

[20] Jindal S.K., Raghuwanshi S.K. (2017). Capacitance and sensitivity calculation of double touch mode capacitive pressure sensor: Theoretical modelling and simulation. Microsyst. Technol..

[21] Ashrafi A., Golnabi H. (1999). A high precision method for measuring very small capacitance changes. Rev. Sci. Instrum..

[22] Jindal S.K., Mahajan A., Raghuwanshi S.K. (2016). A complete analytical model for clamped edge circular diaphragm non-touch and touch mode capacitive pressure sensor. Microsyst. Technol..

[23] Cheng S., Zheng Z. (2003). Large deformation of circular membrane under the concentrated force. Appl. Math. Mech..

[24] Chien W.Z., Wang Z., Xu Y., Chen S. (1981). The symmetrical deformation of circular membrane under the action of uniformly distributed loads in its central portion. Appl. Math. Mech..

